# Comparative efficacy and safety profile of once-weekly Semaglutide versus once-daily Sitagliptin as an add-on to metformin in patients with type 2 diabetes: a systematic review and meta-analysis

**DOI:** 10.1080/07853890.2023.2239830

**Published:** 2023-07-27

**Authors:** Tirath Patel, Fnu Nageeta, Rohab Sohail, Tooba Shaukat Butt, Shyamala Ganesan, Fnu Madhurita, Muhammad Ahmed, Mahrukh Zafar, Wirda Zafar, Mohammad Uzair Zaman, Giustino Varrassi, Mahima Khatri, Satesh Kumar

**Affiliations:** a Medicine, American University of Antigua, Antigua and Barbuda; b Medicine, Ghulam Muhammad Mahar Medical College, Sukkur, Pakistan; c Medicine, Quaid-e-Azam Medical College Bahawalpur, Pakistan, Pakistan; d Medicine, United Medical and Dental College, Karachi, Pakistan; e Medicine, Chanda Medical College, Larkana, Pakistan; f Medicine, American University of the Carribean, United States of America; g Medicine, University of Medicine and health sciences, St. Kitts, Carribean, United States of America; h Medicine, Bacha Khan Medical College, Mardan, Pakistan; i Anesthesiology, Paolo Procacci Foundation, Rome, Italy; j Medicine, Dow University of Health Sciences, Karachi, Pakistan; k Medicine, Shaheed Mohtarma Benazir Bhutto Medical College, Karachi, Pakistan

**Keywords:** Semaglutide, sitagliptin, once-weekly, once daily, type 2 diabetes mellitus, meta-analysis

## Abstract

**Background:**

The emergence of genetically-modified human proteins and glucagon-like peptide-1 (GLP-1) receptor agonists have presented a promising strategy for effectively managing diabetes. Due to the scarcity of clinical trials focusing on the safety and efficacy of semaglutide as an adjunctive treatment for patients with type 2 diabetes who had inadequate glycemic control with metformin, we conducted a systematic review and meta-analysis. This was necessary to fill the gap and provide a comprehensive assessment of semaglutide compared to sitagliptin, a commonly prescribed DPP-4 inhibitor, in this patient population.

**Methods:**

A comprehensive and systematic search was carried out on reputable databases including PubMed, the Cochrane Library, and Elsevier’s ScienceDirect to identify relevant studies that compared the efficacy of once-weekly Semaglutide with once-daily Sitagliptin in individuals diagnosed with type 2 diabetes mellitus. The analysis of the gathered data was performed utilizing the random-effects model, which considers both within-study and between-study variations.

**Results:**

The meta-analysis incorporated three randomized controlled trials (RCTs), encompassing 2401 participants, with a balanced distribution across the treatment groups. The primary focus of the study revolved around evaluating changes in HbA1C, blood pressure, pulse rate, body weight, waist circumference, and BMI. The findings revealed that once-weekly Semaglutide showed substantially improved HbA1C (WMD: −0.98; 95% CI: −1.28, −0.69, p-value: < 0.0001; I2: 100%), systolic (WMD: −3.73; 95% CI: −5.42, −2.04, p-value: <0.0001; I2: 100%) and diastolic blood pressures (WMD: −0.66; 95% CI: −1.02, −0.29, p-value: 0.0005; I2: 100%), and body weight (WMD: −3.17; 95% CI: −3.84, −2.49, p-value: <0.00001; I2: 100%) compared to once-daily Sitagliptin. However, there was an observed increase in pulse rate (WMD: 3.33; 95% CI: 1.61, 5.06, p-value: <0.00001; I2: 100%) associated with Semaglutide treatment. Regarding secondary outcomes, there was an elevated risk of total adverse events and premature treatment discontinuation with Semaglutide. The risk of serious, severe, moderate, and mild adverse events did not significantly differ between the two treatments.

**Conclusions:**

In conclusion, the administration of once-weekly Semaglutide exhibited a substantial reduction in HbA1c, average systolic blood pressure (SBP), mean diastolic blood pressure (DBP), body weight, waist circumference, body mass index (BMI), and a rise in pulse rate, as opposed to the once-daily administration of Sitagliptin.

## Introduction

**Figure 1.**
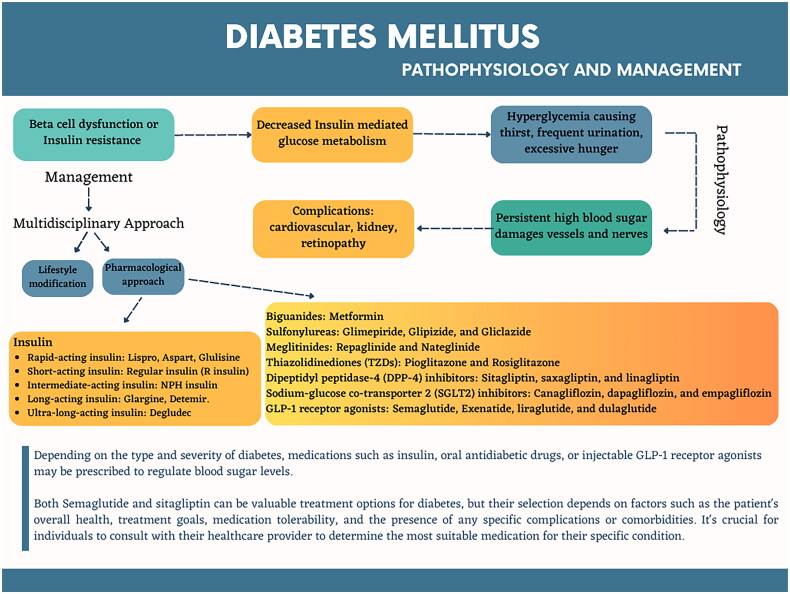
Prisma flow chart; flowchart illustrating the selection process of articles included in the meta-analysis, from an initial 25 articles identified, to the final inclusion of 3 randomized controlled trials (RCTs) after eliminating duplicates, assessing titles and abstracts, and considering only comparative studies for the analysis.

Diabetes mellitus, a spectrum of metabolic disorders, has emerged as a global challenge affecting people worldwide. Interestingly, it is often referred to as a ‘lifestyle disorder,’ indicating its strong connection to our modern way of living [1]. It is a multifaceted and gradual disease; despite the numerous treatment options accessible, a large number of patients with type 2 diabetes fail to reach the suggested blood glucose levels (HbA1c <7·0% [53·0 mmol/mol]; HbA1c ≤6·5% [48·0 mmol/mol]) [2]. While treating diabetes can be challenging, it is not without hope. Clinical practice recommendations are promptly revised based on growing knowledge regarding many facets of diabetes management. The landscape of treatment approaches is evolving, particularly as a result of some groundbreaking clinical trials on more recent anti-diabetic drugs [3]. According to the latest data from the World Health Organization (WHO), the prevalence of diabetes among adults aged 18 years and older was 8.5% in 2014. In 2019, diabetes accounted for 1.5 million deaths directly, with 48% occurring in individuals under 70. Furthermore, diabetes was responsible for an additional 460,000 deaths related to kidney disease. Raised blood glucose levels also contributed to approximately 20% of cardiovascular deaths. These statistics underscore the significant impact of diabetes on global health and highlight the need for effective prevention, management, and increased awareness surrounding this condition [4]. The American Diabetes Association (ADA) has placed significant emphasis on tailoring diabetes management approaches to each patient’s unique circumstances, leveraging technology where appropriate, and prioritizing the prevention of complications [5]. Individualized treatment strategies, such as medication, are required when diet and exercise alone are unable to maintain long-term glycaemic control in persons with type 2 diabetes (T2D) [5]. Despite the availability of many different treatments, maintaining glycemic control in clinical settings without experiencing negative outcomes like hypoglycemic episodes is still exceedingly difficult [6]. Conventional synthetic drugs, combined with the principles of naturopathy, offer a promising approach to managing this condition. This holistic combination can empower individuals to take charge of their health and make positive lifestyle choices, ultimately contributing to the effective management of diabetes. By embracing both modern medicine and natural therapies, individuals with diabetes have the opportunity to navigate their condition and lead fulfilling lives [1]. Figure 1 illustrates the pathophysiology of diabetes and the comprehensive approach employed to maintain healthy blood sugar levels, prevent complications, and promote overall well-being in patients with diabetes.

The development of genetically-engineered human proteins and glucagon-like peptide-1 (GLP-1) receptor agonists have emerged as a promising approach for effectively managing diabetes. The ability to reduce HbA1c levels and body weight while avoiding the risk of hypoglycemia has made this treatment approach particularly valuable for addressing the needs of obese individuals with type 2 diabetes. Nevertheless, the degradation of these proteins by Dipeptidyl Peptidase-4 (DPP-4) and Neutral Endopeptidase represents a significant challenge, as it limits their effectiveness by reducing their half-life to only 1-2 min [7]. Sitagliptin, a DPP-4 inhibitor, is an oral antihyperglycemic agent used for treating T2D in adults in many countries. Sitagliptin inhibits DPP-4, leading to stabilization of the short-lived incretin peptides GLP-1 and GIP. The use of DPP-4 inhibitors in managing hyperglycemia in adults with T2D varies based on local practice guidelines [8]. Both the GLP-1 receptor agonists and DPP-4 inhibitors have the potential to serve as second-line treatments in situations where initial treatments (primarily metformin) fail to achieve glycemic control [9]. The efficacy and safety of semaglutide compared to various comparators, including sitagliptin, have been assessed in six global phase III clinical trials called the ‘Semaglutide Unabated Sustainability in Treatment of Type 2 Diabetes’ (SUSTAIN) program. These trials also evaluated cardiovascular outcomes [10].

We conducted a systematic review and meta-analysis because there is a limited number of clinical trials with a restricted scope that assessed the safety and efficacy of semaglutide compared to sitagliptin, a frequently prescribed DPP-4 inhibitor, as adjunctive therapy for patients with type 2 diabetes who had suboptimal glycemic control with metformin. To our understanding, this is the initial meta-analysis to compare semaglutide 0.5 mg and 1.0 mg with the standard 100 mg dose of sitagliptin to establish the superiority of both options as second-line therapy.

## Methods

In terms of methodology, this meta-analysis adheres to the established guidelines outlined by the Preferred Reporting Items for Systematic Review and Meta-analysis (PRISMA) [11].

### Data sources and search strategy

PubMed, the Cochrane Library and Elsevier’s ScienceDirect databases were systematically searched for clinical studies until April 2023 without language limitations. To gather relevant literature, a combination of specific keywords and medical subject headings (MeSH) such as ‘semaglutide,’ ‘sitagliptin,’ ‘once weekly,’ ‘once daily,’ ‘randomized controlled trial,’ and ‘safety and efficacy’ was utilized. Boolean operators (AND, OR) were employed in the search terms to ensure the inclusion of relevant studies. Supplementary Table S1 provides detailed information about the search methodology. The PICO (population, intervention, comparison, outcome) methodology was adapted with certain modifications. The study population comprised patients with type 2 diabetes. Three researchers (MK, SK, and KM) independently assessed the titles and abstracts of potentially eligible studies to ensure accuracy.

## Inclusion and exclusion criteria

### Inclusion criteria

The study selection criteria for this research involved several essential factors. Firstly, double-blind, randomized controlled trials (RCTs) were considered, emphasizing the use of rigorous scientific methodology to minimize bias. Secondly, a comparison was made between the effects of semaglutide once weekly and sitagliptin once daily, enabling a comprehensive evaluation of their respective impacts. The study’s target population comprised individuals aged 18 and above, with a specific focus on adult patients. Moreover, the inclusion criteria required that the studies involved human participants diagnosed with type 2 diabetes, ensuring direct relevance to the research question. Another crucial aspect was that the selected studies had to report safety and efficacy outcomes, providing valuable insights into the treatments’ effectiveness and any potential associated risks. Lastly, the articles considered for inclusion were explicitly limited to those published in English, ensuring that they were accessible and understandable to the intended readership.

### Exclusion criteria

The study implemented the following criteria for exclusion. Firstly, nonclinical studies were excluded from consideration. Secondly, studies without controls were also excluded. Additionally, observational studies were not considered, including cohort, case-control, cross-sectional case reports, case series studies, editorials, review articles, and conference abstracts. Furthermore, studies with a sample size smaller than 20 were excluded. Finally, any studies with equivocal results were also excluded from the analysis.

### Data extraction

The information from each research study was systematically gathered and recorded using a standardized data collection template. This comprehensive data included details such as the author’s name, publication year, country of origin, study population, participants’ demographic information, medications taken before enrollment in the trial, and clinical outcomes.

The primary outcomes assessed in the studies encompassed several factors, namely the alteration in glycated hemoglobin (HbA1c) levels, changes in both systolic (SBP) and diastolic (DBP) blood pressures, fluctuations in pulse rate, shifts in body weight, variations in waist circumference, and modifications in body mass index (BMI). These outcomes were measured in terms of percentage, millimeters of mercury (mmHg), beats per minute, kilograms (kg), centimeters (cm), and kilograms per square meter (kg/m^2^), respectively.

In addition to the primary outcomes, the studies also examined various secondary outcomes, which included the total occurrence of adverse events (AEs) and the severity levels of these events categorized as serious, severe, moderate, or mild. Furthermore, the number of patients who prematurely discontinued the treatment was also recorded and analyzed.

### Quality assessment of the included studies

The quality assessment of all randomized controlled trials (RCTs) included in the study was conducted by employing the Cochrane risk of bias tool [12].

### Data analysis

The statistical analysis for this meta-analysis was performed using Review Manager version 5.4.1, a tool developed by the Nordic Cochrane Center in collaboration with the Cochrane Collaboration in Denmark in 2014. Only comparative studies were considered in the analysis. The outcomes were presented using forest plots, illustrating the combined effect sizes of relative risks (RRs) for dichotomous outcomes and weighted mean differences (WMDs) for continuous outcomes. A random-effects model with generic-inverse variance was employed to ensure the accuracy of the results.

To assess the potential presence of publication bias, funnel plots were generated for each primary outcome. Higgin’s I2 test was employed to evaluate the level of heterogeneity, classified as low, moderate, or high. In instances where significant heterogeneity (> 75%) [13] was observed, a sensitivity analysis was conducted by systematically excluding individual studies to investigate their impact on the overall findings.

For all analyses, a p-value less than 0.05 was considered statistically significant. The authors thoroughly examined the data to guarantee the precision and dependability of their findings.

Given that the data in this study were collected from previous clinical trials where participants had already provided informed consent, ethical approval from a research ethics committee was not required for this investigation.

## Results

### Eligible studies

**Figure 2.**
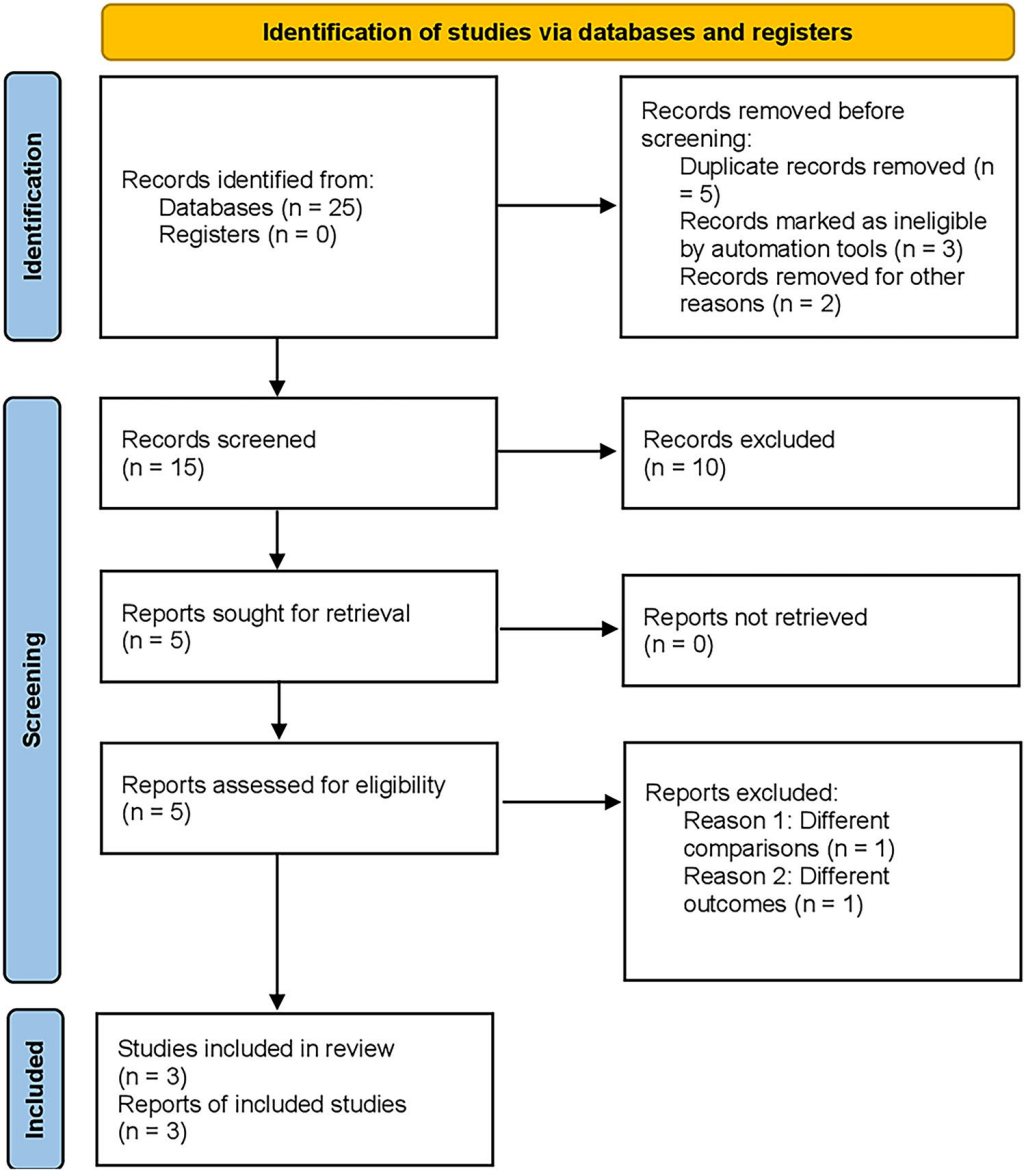
Forest plot of change in HbA1C (Glycated hemoglobin); the figure provides a comparison of the effects of once-weekly Semaglutide treatment and once-daily Sitagliptin on HbA1c values. The analysis demonstrates that once-weekly Semaglutide treatment leads to a significant decrease in HbA1c values compared to once-daily Sitagliptin. Notably, the 1 mg dose of Sitagliptin exhibits a more substantial reduction in HbA1c values; WMD- weighted mean difference; CI- Confidence interval; M-H- Mantel Hansel.

The preliminary output of the literature review consisted of 25 articles, which were subsequently refined by eliminating duplicates and assessing the titles and abstracts. From this process, three randomized controlled trials (RCTs) were identified [10,14,15]. Only comparative studies were considered for inclusion in the meta-analysis, and a detailed depiction of the search methodology is presented in Figure 2 using the PRISMA diagram. All three RCTs were multicenter comparative studies, with two of them being double-blind and one being open-label [10]. The mean duration of follow-up was 43 weeks, and the collection of articles included in this study ranged from the years 2017 to 2021.

### Baseline characteristics of the included patients

This meta-analysis comprised a total of 2401 participants, with 800 (33.3%) subjects assigned to the Semaglutide 0.5 mg group, 801 (33.4%) to the Semaglutide 1 mg group, and 800 (33.3%) to the Sitagliptin 100 mg group. The gender distribution of the participants showed that 1199 (49.9%) were male and 1096 (45.6%) were female. The average age of the participants was 55.4 ± 10.4 years. The mean HbA1C was 8.1 ± 0.9%, and the mean duration of diabetes was 7 ± 5.2 years. Most of the participants were overweight, with an average BMI of 28.6 ± 4. Prior to enrollment in the respective trials, 1690 out of 2401 (70.4%) participants were taking biguanides, while others were on medications such as sulphonylureas, thiazolidinediones, DPP-4 inhibitors, and alpha-glucosidase inhibitors. A detailed summary of the baseline characteristics of the participants can be found in Tables 1 and 2.

**Table 1.** Baseline characteristics of the included participants.

			No. of participants			Age, years (mean ± SD)			Males No. (%)			Females No. (%)		
Study and year	Study design	Total No. of participants	Semaglutide 0.5 mg	Semaglutide 1.0 mg	Sitagliptin 100 mg	Semaglutide 0.5 mg	Semaglutide 1.0 mg	Sitagliptin 100 mg	Semaglutide 0.5 mg	Semaglutide 1.0 mg	Sitagliptin 100 mg	Semaglutide 0.5 mg	Semaglutide 1.0 mg	Sitagliptin 100 mg
Ahrén (2017) [14]	RCT	1225	409	409	407	54.8 ± 10.2	56.0 ± 9.4	54.6 ± 10.4	209 (51)	205 (50)	208 (50)	200 (49)	204 (50)	199 (49)
Seino (2018) [10]	RCT	308	103	102	103	58.8 ± 10.4	58.1 ± 11.6	57.9 ± 10.1	207 (76.7)	205 (73.5)	208 (78.6)	202 (23.3)	204 (26.5)	199 (21.4)
Linong (2021) [15]	RCT	868	288	290	290	53.0 ± 11.4	53.0 ± 10.6	53.1 ± 10.4	160 (55.6)	154 (53.1)	185 (63.8)	128 (44.4)	136 (46.9)	105 (36.2)

SD: standard deviation; RCT: randomized controlled trial.

**Table 2.** Baseline Demographics of the included participants.

	HbA1c, % (mean ± SD)			FPG, mmol/L (mean ± SD)			BMI, kg/m2 (mean ± SD)			Diabetes duration, years (mean ± SD)			Body weight, kg (mean ± SD)		
Study and year	Semaglutide 0.5 mg	Semaglutide 1.0 mg	Sitagliptin 100 mg	Semaglutide 0.5 mg	Semaglutide 1.0 mg	Sitagliptin 100 mg	Semaglutide 0.5 mg	Semaglutide 1.0 mg	Sitagliptin 100 mg	Semaglutide 0.5 mg	Semaglutide 1.0 mg	Sitagliptin 100 mg	Semaglutide 0.5 mg	Semaglutide 1.0 mg	Sitagliptin 100 mg
Ahrén (2017) [14]	8.0 ± 0.9	8.0 ± 0.9	8.2 ± 0.9	9.3 ± 2.4	9.3 ± 2.2	9.6 ± 2.2	32.4 ± 6.2	32.5 ± 6.6	32.5 ± 5.8	6.4 ± 4.7	6.7 ± 5.6	6.6 ± 5.1	89.9 ± 20.4	89.2 ± 20.7	89.3 ± 19.7
Seino (2018) [10]	8.2 ± 1.0	8.0 ± 0.9	8.2 ± 0.9	9.2 ± 2.1	9.2 ± 1.8	9.5 ± 2.0	25.1 ± 3.8	26.1 ± 5.2	25.1 ± 3.6	8.0 ± 5.2	7.8 ± 6.9	8.1 ± 6.7	67.8 ± 11.7	70.8 ± 16.4	69.4 ± 12.9
Linong (2021) [15]	8.1 ± 0.9	8.1 ± 0.9	8.1 ± 0.9	9.30 ± 2.67	9.29 ± 2.22	9.05 ± 2.21	28.2 ± 5.0	27.9 ± 5.0	27.3 ± 4.7	6.3 ± 5.4	6.7 ± 4.9	6.1 ± 5.2	77.6 ± 16.4	76.1 ± 16.3	75.5 ± 14.7

SD: Standard deviation; HbA1C: glycated hemoglobin; FPG: Fasting plasma glucose; BMI: body mass index.

### Quality assessment and publication bias

Through the implementation of the Cochrane methodology for evaluating RCTs (Supplemental Figure 1), trials of medium to high quality were identified. Moreover, based on the funnel plots, it was determined that the results were not affected by publication bias (Supplemental Figure 2).

### Primary outcomes

The primary outcomes comprised of change in HbA1C, change in mean systolic and diastolic blood pressures, change in pulse rate, change in weight, change in BMI, and change in waist circumference.

### Change in HbA1C

**Figure 3.**
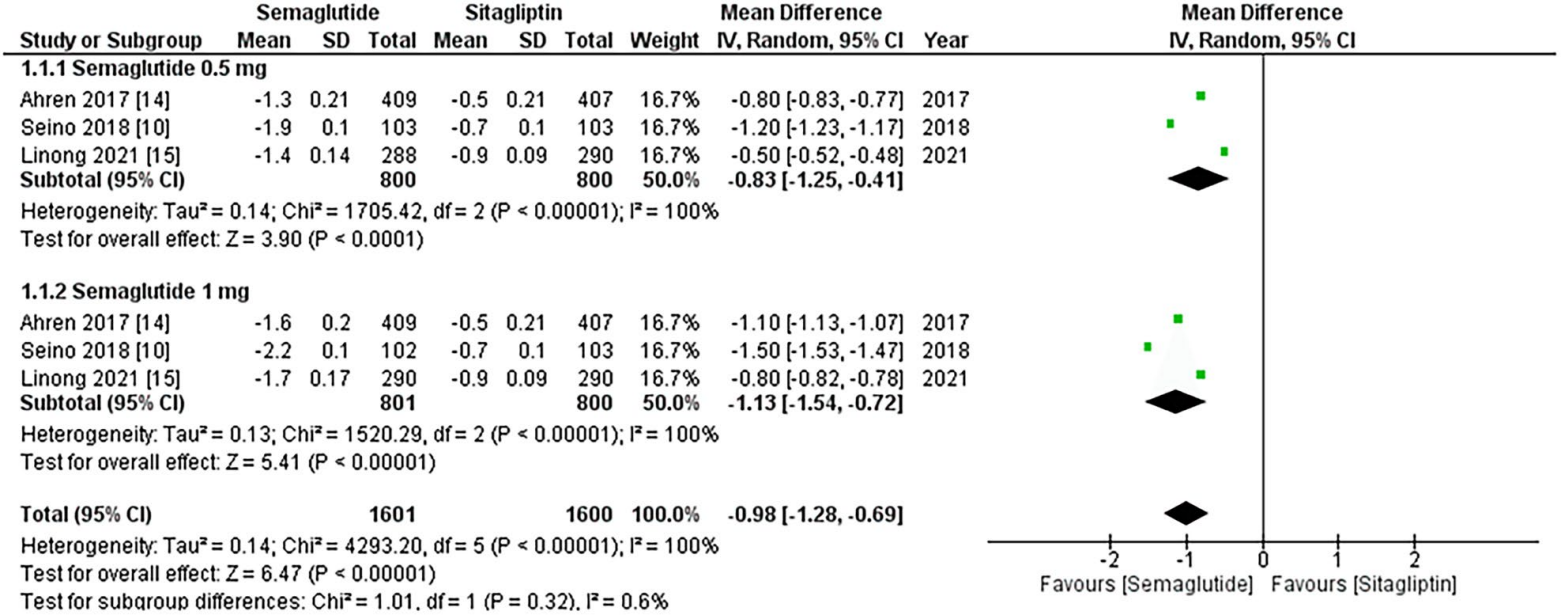
Forest plot of change in mean SBP (systolic blood pressure); the Figure demonstrates a pooled analysis comparing once-weekly Semaglutide and once-daily Sitagliptin, revealing a significant reduction in mean SBP with Semaglutide treatment compared to Sitagliptin. The decrease is more noticeable when the 1 mg dose of Sitagliptin is taken into account; WMD- weighted mean difference; CI- Confidence interval; M-H- Mantel Hansel.

All three studies reported change in HbA1c levels, and upon pooled analysis, it was found that once-weekly Semaglutide treatment resulted in a significant decrease in HbA1c values after treatment (WMD: −0.98; 95% CI: −1.28, −0.69, p-value: < 0.0001; I2: 100%) compared to once daily Sitagliptin, particularly the 1 mg dose (WMD: −1.13; 95% CI: −1.54, −0.72, p-value: <0.00001; I2: 100%) as shown in Figure 3. A leave-one-out sensitivity analysis was conducted to investigate the high heterogeneity observed among the studies. It was determined that the heterogeneity was not due to any specific study.

### Changes in SBP and DBP

**Figure 4.**
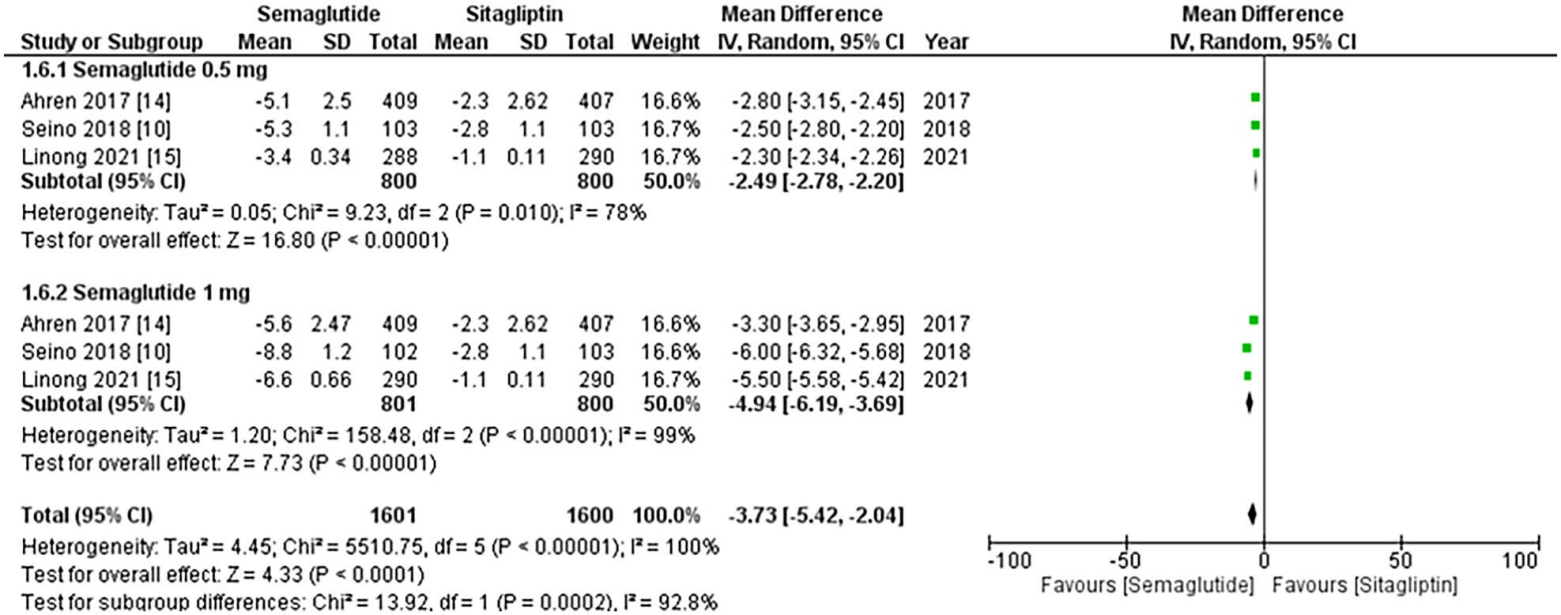
Forest plot of change in mean DBP (Diastolic blood pressure); the figure demonstrates a significant decrease in mean DBP with once-weekly Semaglutide compared to once-daily Sitagliptin, with greater reduction observed for the 1 mg dose; WMD- weighted mean difference; CI- Confidence interval; M-H- Mantel Hansel.

**Figure 5.**
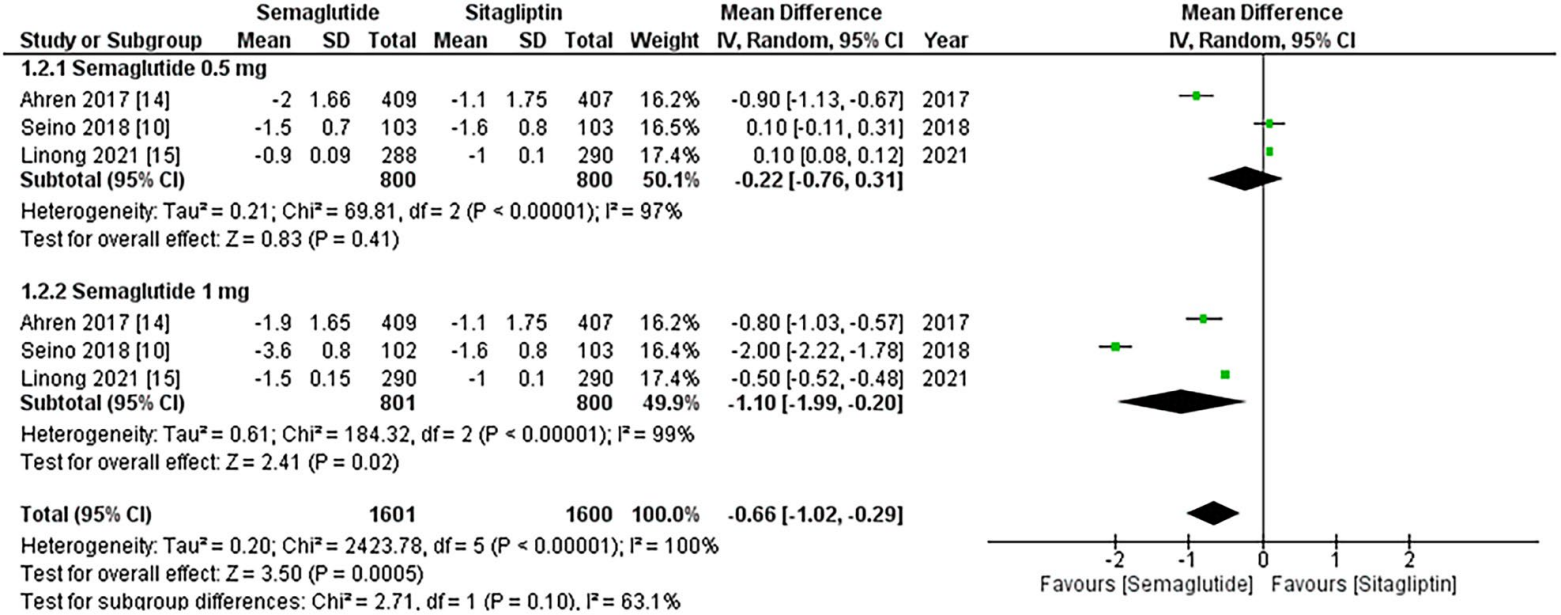
Forest plot of change in pulse rate; the figure demonstrates a significant increase in pulse rate associated with once-weekly Semaglutide treatment compared to once-daily Sitagliptin. Furthermore, the increase in pulse rate was more pronounced in patients taking the 1 mg once-weekly dose of Semaglutide; WMD- weighted mean difference; CI- Confidence interval; M-H- Mantel Hansel.

The results from all three studies included the SBP and DBP values, and after conducting a pooled analysis, it was found that treatment with once-weekly Semaglutide resulted in a significant reduction in the mean SBP (WMD: −3.73; 95% CI: −5.42, −2.04, p-value: <0.0001; I2: 100%) compared to once-daily Sitagliptin, which was more noticeable with the 1 mg dose (WMD: −4.94; 95% CI: −6.19, −3.69, p-value: <0.00001; I2: 100%) as shown in Figure 4. Similarly, the pooled analysis for DBP showed a significant decrease in mean DBP (WMD: −0.66; 95% CI: −1.02, −0.29, p-value: 0.0005; I2: 100%) with the use of once-weekly Semaglutide compared to once-daily Sitagliptin. This reduction was more pronounced with the 1 mg dose (WMD: −1.10; 95% CI: −1.99, −0.20, p-value: <0.00001; I2: 99%) as shown in Figure 5. A leave-one-out sensitivity analysis was performed to address the high heterogeneity in the results of these two outcomes, which determined that any specific study did not cause heterogeneity.

### Change in pulse rate

**Figure 6.**
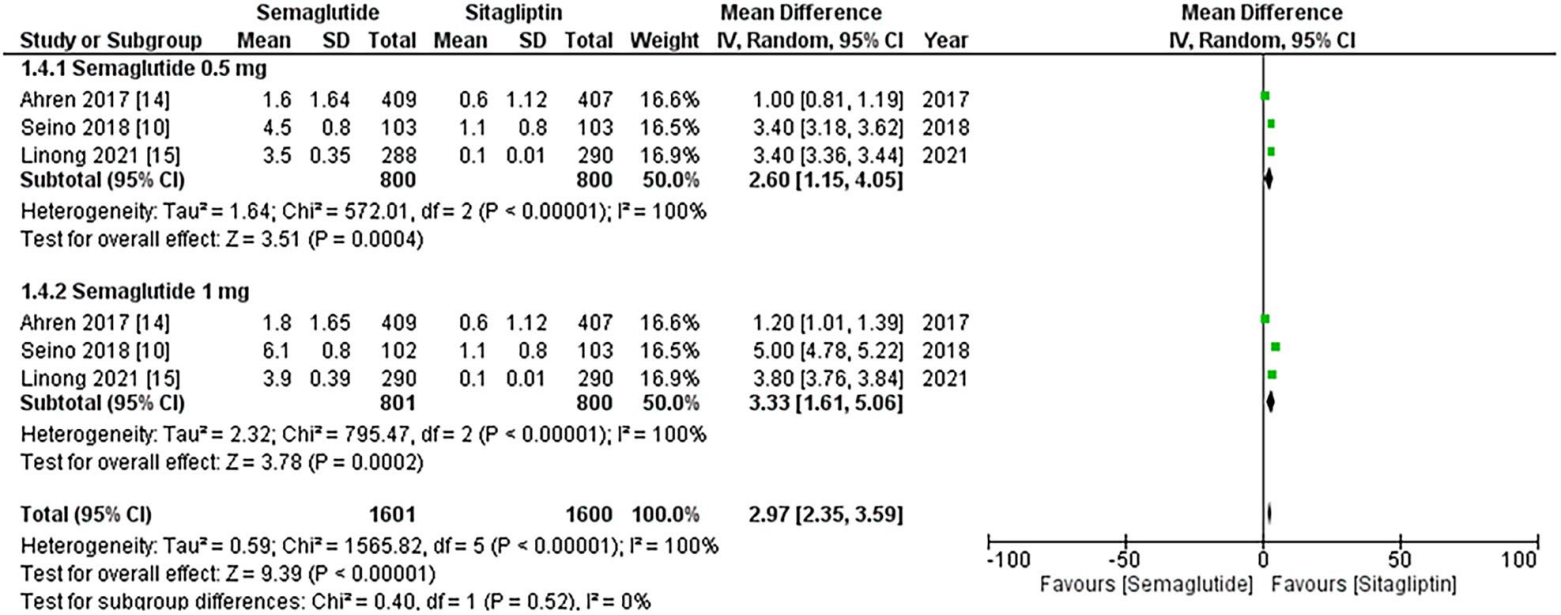
Forest plot of change in body weight; the figure shows a significant decrease in body weight after treatment with once-weekly Semaglutide compared to once-daily Sitagliptin. Additionally, the 1 mg dose of Semaglutide exhibited a more remarkable reduction in body weight than the 0.5 mg dose; WMD- weighted mean difference; CI- Confidence interval; M-H- Mantel Hansel.

All three studies included pulse rate values, and the pooled analysis revealed a significant increase in pulse rate associated with once-weekly Semaglutide treatment (WMD: 2.97; 95% CI: 2.35, 3.59, p-value: <0.00001; I2: 100%) compared to once-daily Sitagliptin. The increase was more pronounced in patients taking the 1 mg once-weekly dose of Semaglutide (WMD: 3.33; 95% CI: 1.61, 5.06, p-value: <0.00001; I2: 100%), as depicted in Figure 6. A leave-one-out sensitivity analysis was conducted to account for the high in-study heterogeneity, and the results indicated that no particular study contributed to the observed heterogeneity.

### Change in body weight, waist circumference, and BMI

**Figure 7.**
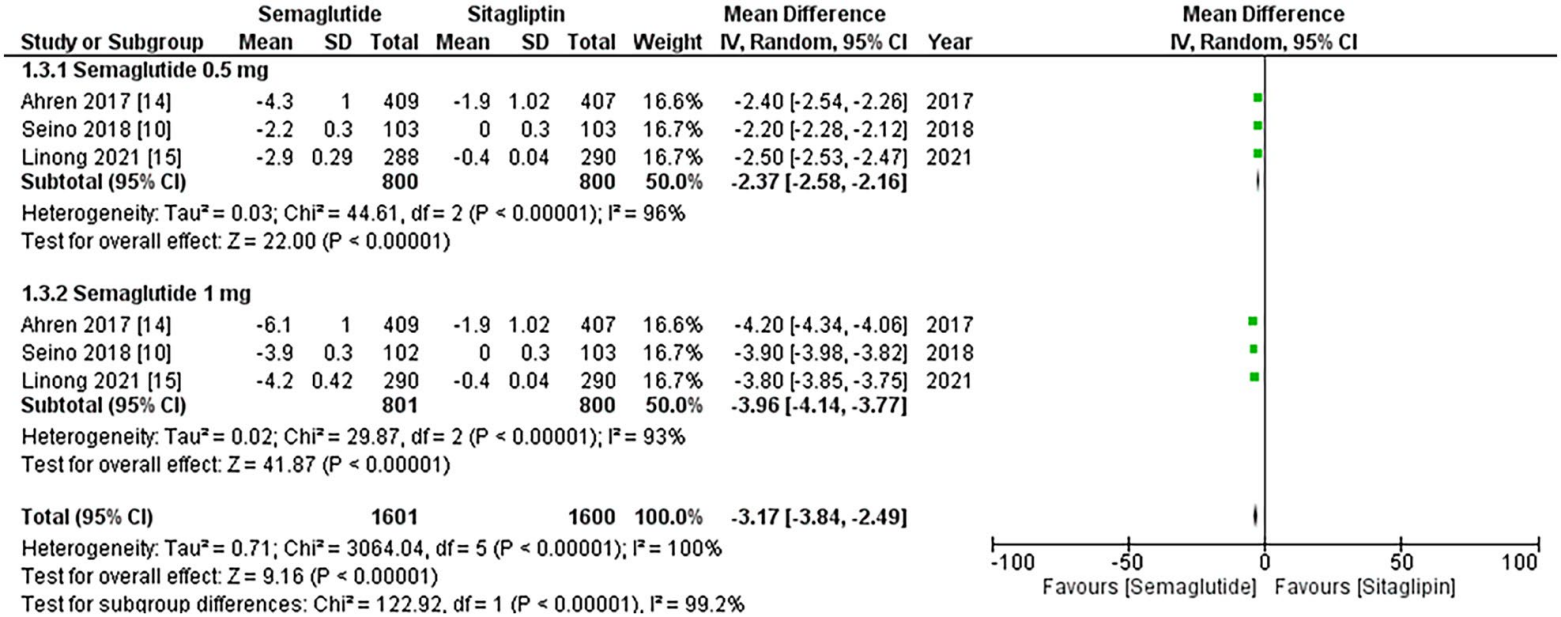
Forest plot of change in waist circumference; the analysis showed waist circumference reduction with once-weekly Semaglutide, Highlighting enhanced effects at 1 mg dose; WMD- weighted mean difference; CI- Confidence interval; M-H- Mantel Hansel.

**Figure 8.**
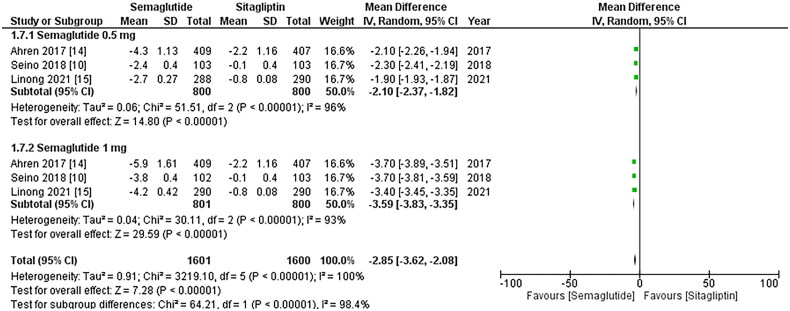
Forest plot of change in Body mass index (BMI); the figure demonstrates a significant decrease in BMI after treatment with the once-weekly Semaglutide regimen compared to the once-daily Sitagliptin regimen. Notably, the 1 mg dose of Semaglutide resulted in a more notable reduction in BMI than the 0.5 mg dose; WMD- weighted mean difference; CI- Confidence interval; M-H- Mantel Hansel.

**Figure 9.**
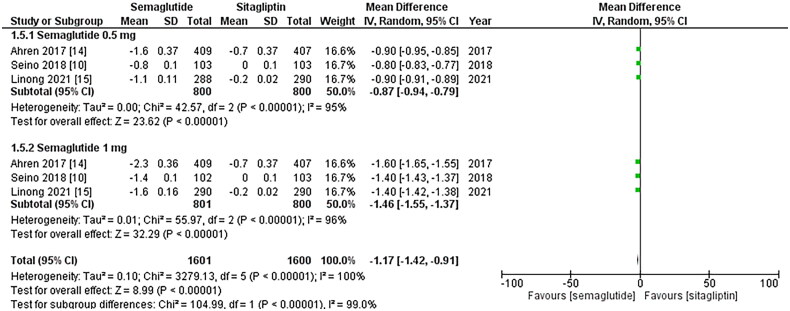
.

The outcomes mentioned above were reported by all three studies, and the pooled analysis revealed that once-weekly Semaglutide treatment led to a significant decrease in body weight after treatment (WMD: −3.17; 95% CI: −3.84, −2.49, p-value: <0.00001; I2: 100%) compared to once-daily Sitagliptin. However, the 1 mg dose of Semaglutide resulted in a more remarkable reduction in body weight than the 0.5 mg dose (WMD: −3.96; 95% CI: −4.14, −3.77, p-value: <0.00001; I2: 93%), as illustrated in Figure 7. Furthermore, treatment with once-weekly Semaglutide was linked to a significant reduction in waist circumference (WMD: −2.85; 95% CI: −3.62, −2.08, p-value: <0.00001; I2: 100%), with the 1 mg dose of Semaglutide showing more noticeable effects (WMD: −3.59; 95% CI: −3.83, −3.35, p-value: <0.00001; I2: 93%), as depicted in Figure 8. Likewise, the once-weekly Semaglutide regimen was associated with a significant decrease in BMI after treatment (WMD: −1.17; 95% CI: −1.42, −0.91, p-value: <0.00001; I2: 100%) compared to the once-daily Sitagliptin regimen. However, the 1 mg dose of Semaglutide resulted in a more notable reduction in BMI than the 0.5 mg dose (WMD: −1.46; 95% CI: −1.55, −1.37, p-value: <0.00001; I2: 96%), as shown in Figure 9. A sensitivity analysis was conducted to account for the high heterogeneity in the outcomes, indicating that no study caused heterogeneity.

### Secondary outcomes

In this study, various adverse effects were examined as secondary outcomes, including different levels of adverse events such as total adverse events, serious adverse events, severe adverse events, moderate adverse events, mild adverse events, and premature treatment discontinuation. For a detailed breakdown of these secondary outcomes, please refer to Table 3. After analyzing the combined data, it was found that using a once-weekly Semaglutide treatment was associated with a significantly higher risk of total adverse events and premature treatment discontinuation. The risk of serious, severe, moderate, and mild adverse events also increased in the group receiving once-weekly Semaglutide, but the results were not statistically significant. Furthermore, when analyzing specific subgroups, it was observed that the risk of premature treatment discontinuation and severe adverse events was more pronounced with the 1 mg dose of Semaglutide, whereas the total, serious, moderate, and mild adverse events were more noticeable in patients who received the 0.5 mg dose of Semaglutide.

**Table 3.** Secondary outcomes.

Outcomes	Effect size (RR or WMD)	95% CI	p-value	I2
Total AE	**RR: 1.05**	**1.00, 1.09**	**0.04**	**0%**
Semaglutide 0.5 mg Vs. Sitagliptin	RR: 1.06	1.00, 1.12	0.07	0%
Semaglutide 1 mg Vs. Sitagliptin	RR: 1.04	0.97, 1.10	0.25	0%
Serious AE	**RR: 1.20**	**0.91, 1.58**	**0.21**	**0%**
Semaglutide 0.5 mg Vs. Sitagliptin	RR: 1.24	0.83, 1.86	0.29	3%
Semaglutide 1 mg Vs. Sitagliptin	RR: 1.16	0.78, 1.72	0.47	0%
Severe AE	**RR: 1.08**	**0.77, 1.53**	**0.64**	**0%**
Semaglutide 0.5 mg Vs. Sitagliptin	RR: 1.07	0.66, 1.76	0.78	0%
Semaglutide 1 mg Vs. Sitagliptin	RR: 1.10	0.67, 1.78	0.71	0%
Moderate AE	**RR: 1.10**	**0.96, 1.28**	**0.18**	**8%**
Semaglutide 0.5 mg Vs. Sitagliptin	RR: 1.15	0.95, 1.38	0.14	0%
Semaglutide 1 mg Vs. Sitagliptin	RR: 1.08	0.81, 1.45	0.60	36%
Mild AE	**RR: 1.05**	**1.00, 1.10**	**0.07**	**0%**
Semaglutide 0.5 mg Vs. Sitagliptin	RR: 1.06	0.99, 1.13	0.12	0%
Semaglutide 1 mg Vs. Sitagliptin	RR: 1.04	0.96, 1.11	0.33	0%
Premature treatment discontinuation	**RR: 3.27**	**2.31, 4.63**	**< 0.00001**	**0%**
Semaglutide 0.5 mg Vs. Sitagliptin	RR: 2.64	1.59, 4.38	0.0002	0%
Semaglutide 1 mg Vs. Sitagliptin	RR: 3.97	2.45, 6.42	< 0.00001	0%

AE: Adverse events; RR: relative risk, CI: confidence interval; I2: heterogeneity.

## Discussion

The continuous increase in the global occurrence of type 2 diabetes has emerged as a significant concern within the healthcare sector, primarily due to the subsequent rise in patient morbidity and mortality resulting from cardiovascular, renal, and neurological complications. Furthermore, the escalating financial and resource expenditures required to handle these complications have added to the gravity of the issue [16]. Two broad categories of incretin therapies have been formulated with the intention of leveraging the antihyperglycemic properties of GLP-1 [17]. Both agents, known as glucagon-like peptide (GLP)-1 receptor agonists and dipeptidyl peptidase-4 (DPP-4) inhibitors, contribute to the increased activation of GLP-1 receptors. GLP-1 receptor agonists deliver significant concentrations of the respective substances in the bloodstream, leading to targeted interaction with GLP-1 receptors. The question remains debatable whether combining GLP-1 receptor agonists and DPP-4 inhibitors would result in a more pronounced reduction in glucose levels compared to using either category of medication independently [18]. A previous analysis conducted by Scheen et al. in 2012 indicated that GLP-1 receptor agonists exhibited greater potency in reducing glucose levels, promoting weight loss, and lowering systolic blood pressure compared to DPP-4 inhibitors [19]. Conversely, DPP-4 inhibitors were found to be more convenient to administer, cost-effective, and better tolerated regarding gastrointestinal effects. However, it is worth noting that the NICE guidelines and analysis did not specifically focus on obese patients. In a separate study in 2017, Li et al. in examined the impact of sitagliptin on obese patients undergoing insulin treatment [20]. They discovered that sitagliptin led to a reduction in body mass index (BMI) and a decrease in hypoglycemic episodes. Similarly, a cohort study by Kodera et al. in 2017 demonstrated the efficacy of sitagliptin in managing glucose metabolic disorders in obese Japanese patients with T2DM [21]. These findings prompt the question of how effective Semaglutide and Sitagliptin are in managing T2DM in patients who are using metformin [22]. Therefore, we conducted a comprehensive review and meta-analysis to assess the effectiveness and hypoglycemic risk of standard doses of Semaglutide (0.5 mg and 1 mg) with 100 mg sitagliptin as an add-on to metformin in patients with T2DM.

This meta-analysis of 3 randomized controlled trials (RCT) with 2401 participants compares the efficacy and safety profile of recently introduced standard doses of Semaglutide (0.5 mg and 1 mg) with 100 mg sitagliptin as an add-on to metformin in patients with T2DM. Our research aimed to address various outcome measures, including alterations in HbA1C levels, modifications in SBP and DBP, shifts in pulse rate, changes in body weight, waist circumference, and BMI. Moreover, our investigation also assessed several secondary outcome measures, encompassing overall adverse events, significant adverse events, severe adverse events, moderate adverse events, mild adverse events, and premature cessation of treatment. The investigation we conducted appraised the effect of Semaglutide therapy on HbA1c values and found a substantial reduction in contrast to Sitagliptin administered once per day. Nonetheless, previous research conducted by BUSE et al. in 2020 indicated a minor rise in HbA1c initially, followed by a decline until it stabilized. This finding challenges the efficacy of Semaglutide. In addition, during the extension, patients who continued with Sitagliptin maintained a constant HbA1c level without any additional decrease achieved after the primary phase [23]. The administration of Semaglutide on a weekly basis led to a notable decrease in the average SBP and DBP. Lavernia et al. in 2020 also reported similar findings, demonstrating that individuals receiving oral semaglutide experienced mean reductions of 2–5 mmHg in systolic blood pressure and 1–2 mmHg in diastolic blood pressure upon completion of the study [24].

In the study conducted by Hong et al.in 2018, the effects of semaglutide on blood pressure control were examined. The results indicated a significant reduction in systolic blood pressure (SBP) when compared to other treatments (weighted mean difference [WMD]: −0.29 mmHg, 95% confidence interval [CI]: −0.65 to 0.07, *p* = 0.113) [25]. However, no significant difference was observed in diastolic blood pressure (DBP) (WMD: −0.29 mmHg, 95% CI: −0.65 to 0.07, *p* = 0.113). On the other hand, semaglutide exhibited a notable decrease in SBP (WMD: −2.55 mmHg, 95% CI: −3.22 to −1.88, *p* < 0.001) compared to other therapies for blood pressure management. Interestingly, the use of semaglutide resulted in a higher pulse rate compared to alternative treatments (WMD: 2.21 bpm, 95% CI: 1.54 to 2.88, *p* < 0.001), although there was moderate heterogeneity observed among the studies [25]. Following the treatment with semaglutide, a significant decline in body weight, waist circumference, and BMI was observed compared to the administration of sitagliptin on a daily basis. The superior efficacy of oral semaglutide in reducing HbA1c levels and body weight, in contrast to sitagliptin, aligns with findings from other comparative trials that have demonstrated the superiority of GLP-1 receptor agonists (GLP-1RAs) over DPP-4 inhibitors in terms of glycemic control and weight reduction. These outcomes achieved with oral semaglutide hold clinical importance since enhanced glycemic control is associated with improved outcomes in diabetes management, and some patients may have a preference for oral medications to attain such enhanced glycemic control [26]. Moreover, significant weight loss, which is clinically important, contributes to improved glycemic control and mitigates cardiovascular risk factors. In a study conducted by Wilding et al. in 2021, the response to Semaglutide in obese patients without diabetes was examined [27]. The study found that when Semaglutide was used as a supplementary treatment alongside lifestyle intervention, adults classified as obese (or overweight with weight-related coexisting conditions) experienced a substantial average weight loss of 14.9% from their initial weight. This weight loss was significantly more significant than that observed in the placebo group combined with lifestyle intervention, exceeding it by an additional 12.4 percentage points. The observed mean weight loss of 14.9% with Semaglutide also exceeded the weight loss range of 4.0 to 10.9% reported with approved antiobesity medications compared to baseline [27]. These findings highlight the positive response to Semaglutide and suggest its potential efficacy in promoting weight loss in obese individuals without diabetes when used in conjunction with lifestyle intervention. A study conducted by Rubino et al. [28] in 2021 found that continuing semaglutide treatment beyond the initial randomized period resulted in sustained and significant weight loss. The weight loss achieved during the run-in period continued and reached a plateau at around week 60 to week 68, resulting in an estimated reduction of 17.4% over the entire trial. In contrast, participants who switched to placebo at week 20 gradually regained weight. The study’s findings highlight the importance of maintaining semaglutide treatment for longer, as it showed more significant benefits compared to switching to placebo. This emphasizes the chronic nature of obesity and the need for remedies that can sustain and maximize weight loss. Furthermore, the sustained weight loss with semaglutide was accompanied by improvements in waist circumference, lipid profiles, and glucose metabolism, which are essential cardiometabolic risk factors. Similar sustained weight loss has been associated with improvements in obesity-related complications, particularly type 2 diabetes, with treatment guidelines recommending a sustained weight loss of 5% to 15% for individuals with these conditions [28]. In a phase 3 trial conducted by Miles et al. [29] in 2018, the efficacy and safety of semaglutide were compared with placebo and other pharmacologic therapy for diabetes (PTD) in multicenter SUSTAIN trials. Semaglutide demonstrated lower hemoglobin A1c (HbA1c) levels by approximately −1.5% and weight reductions of approximately −4.5 kg, comparable to dulaglutide’s effects on HbA1c lowering. Additionally, semaglutide showed significant cardiovascular outcomes, including a reduced risk of death from cardiovascular causes, nonfatal myocardial infarction, or nonfatal stroke, with a hazard ratio of 0.74 and a 95% confidence interval of 0.58–0.95. These findings highlight the efficacy of semaglutide in improving glycemic control, promoting weight loss, and reducing cardiovascular risks in patients with diabetes [29].

To ensure the accurate interpretation and applicability of our meta-analysis findings, it is crucial to consider and evaluate the presence of substantial heterogeneity thoroughly. Various methodologies, such as leave-one-out analysis, were utilized to investigate and elucidate the sources of heterogeneity while conducting sensitivity analyses to ascertain the robustness of the results. Potential factors contributing to heterogeneity may encompass dissimilarities in study design, including variances in participant characteristics, outcome measurements, and durations of follow-up. Methodological disparities and variations in clinical and demographic attributes could also contribute to the observed heterogeneity. Even when the included studies share similarities in design and characteristics, statistical heterogeneity can arise due to random fluctuations in the results. Statistical tests, such as the I-squared (I^2^) statistic or Cochran’s Q test, are commonly employed to assess the presence of statistical heterogeneity.

It is important to note some limitations in our study. One limitation that can affect the validity of a study is the need for adequate sample size and a limited number of trials conducted. When the sample size is small, and the number of attempts is insufficient, it can lead to reduced statistical power and compromise the reliability of the findings. Secondly, Possible variations in ethnic backgrounds, as well as differences in the initial characteristics of the participants, may have played a role in the clinical heterogeneity observed. Furthermore, a limited number of studies provided information on various treatments besides metformin that patients were utilizing. Consequently, incorporating additional therapies like Diet and exercise therapy, sulphonylurea, α-glucosidase inhibitor, and thiazolidinediones, which might influence the outcomes, was not adequately reported in most studies.

## Conclusion

In summary, the administration of once-weekly Semaglutide led to a significant reduction in HbA1c, average systolic blood pressure (SBP), mean diastolic blood pressure (DBP), body weight, waist circumference, and BMI, as well as an increase in pulse rate, compared to the once-daily administration of Sitagliptin. Moreover, Semaglutide demonstrated a favorable safety profile similar to other GLP-1 receptor agonists. Based on these findings, we conclude that once-weekly Semaglutide shows great promise as an adjunctive treatment to metformin when monotherapy fails to achieve adequate glycemic control in individuals with type 2 diabetes.

## Supplementary Material

Supplemental Material

Supplemental Material

## Data Availability

Data Availability Statement: The data supporting the findings of this study, titled ‘ Comparative Efficacy and Safety Profile of once-weekly Semaglutide versus once-daily Sitagliptin as an add-on to Metformin in Patients with Type 2 Diabetes: A Systematic Review and Meta-Analysis’ are available on PubMed and can be accessed *via* their respective DOI (Digital Object Identifier). Interested researchers can retrieve the data by searching for the article using the provided DOI. Should additional information or data be required for the purpose of verification, replication, or further analysis, the corresponding authors of the study can be contacted. Their contact details are as follows: Tirath Patel Institute: American University of Antigua Email: Tirathp611@mgail.com contact: 091-8128250661 Department: Medicine country: Antigua and Barbuda The authors are committed to promoting transparency and facilitating scientific progress. While the primary data are available on PubMed, the corresponding authors are willing to assist researchers in accessing any additional information that may be necessary to support their work.
