# Supplementary material for: Comparative efficacy and safety profile of once-weekly Semaglutide versus once-daily Sitagliptin as an add-on to metformin in patients with type 2 diabetes: a systematic review and meta-analysis
Source: Ann Med. 2023 Jul 27;55(2):2239830. doi: 10.1080/07853890.2023.2239830 (PMC10375936; doi:10.1080/07853890.2023.2239830)
Supplement: Supplemental Material [file IANN_A_2239830_SM6261.docx]

**Figure S1: Quality assessment of the included randomized controlled trials; The Cochrane method for evaluating randomized controlled trials was employed to identify trials of medium to high quality.**


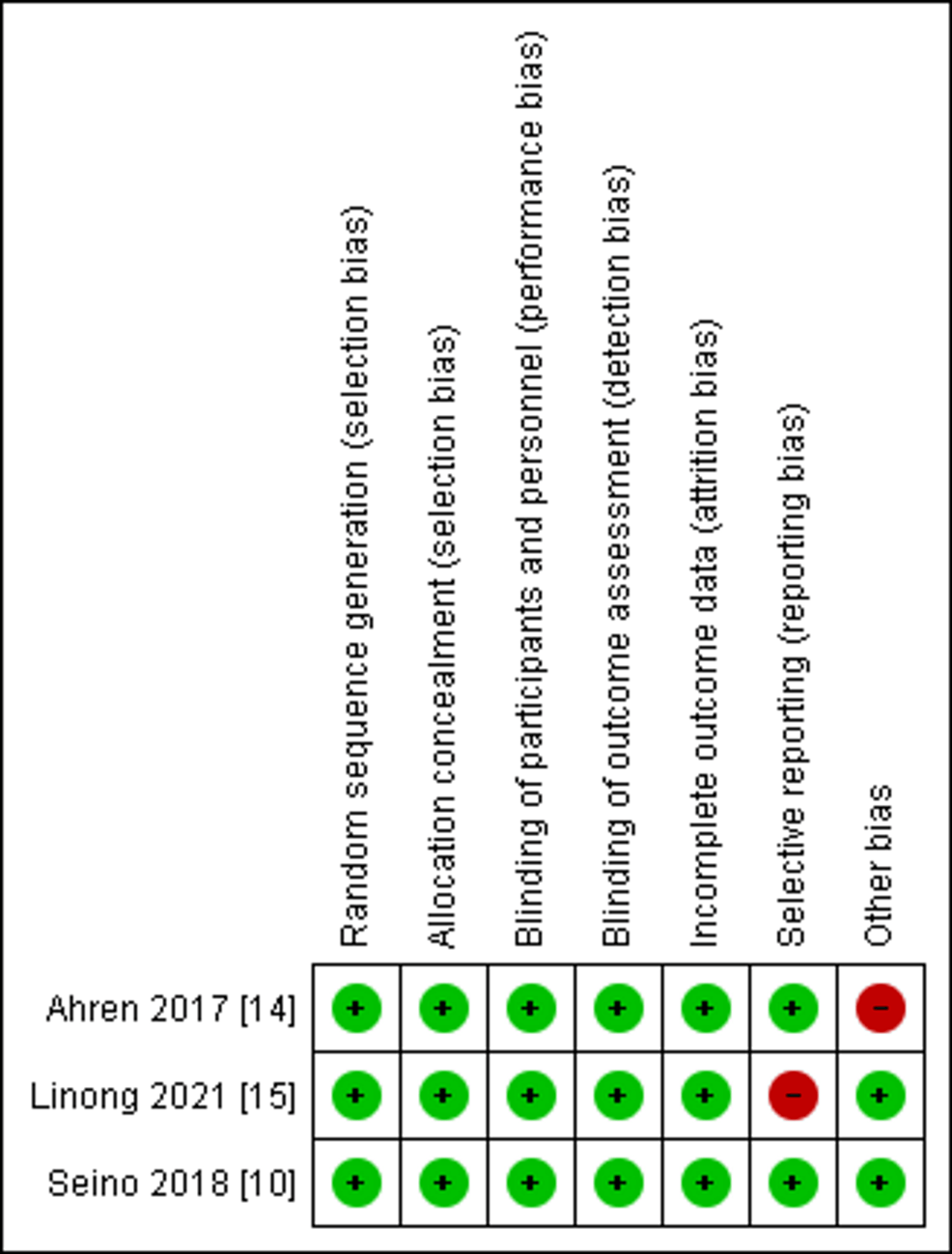


**Figure S2: Funnel plots: (A) Change in HbA1C (B) Changes in SBP**


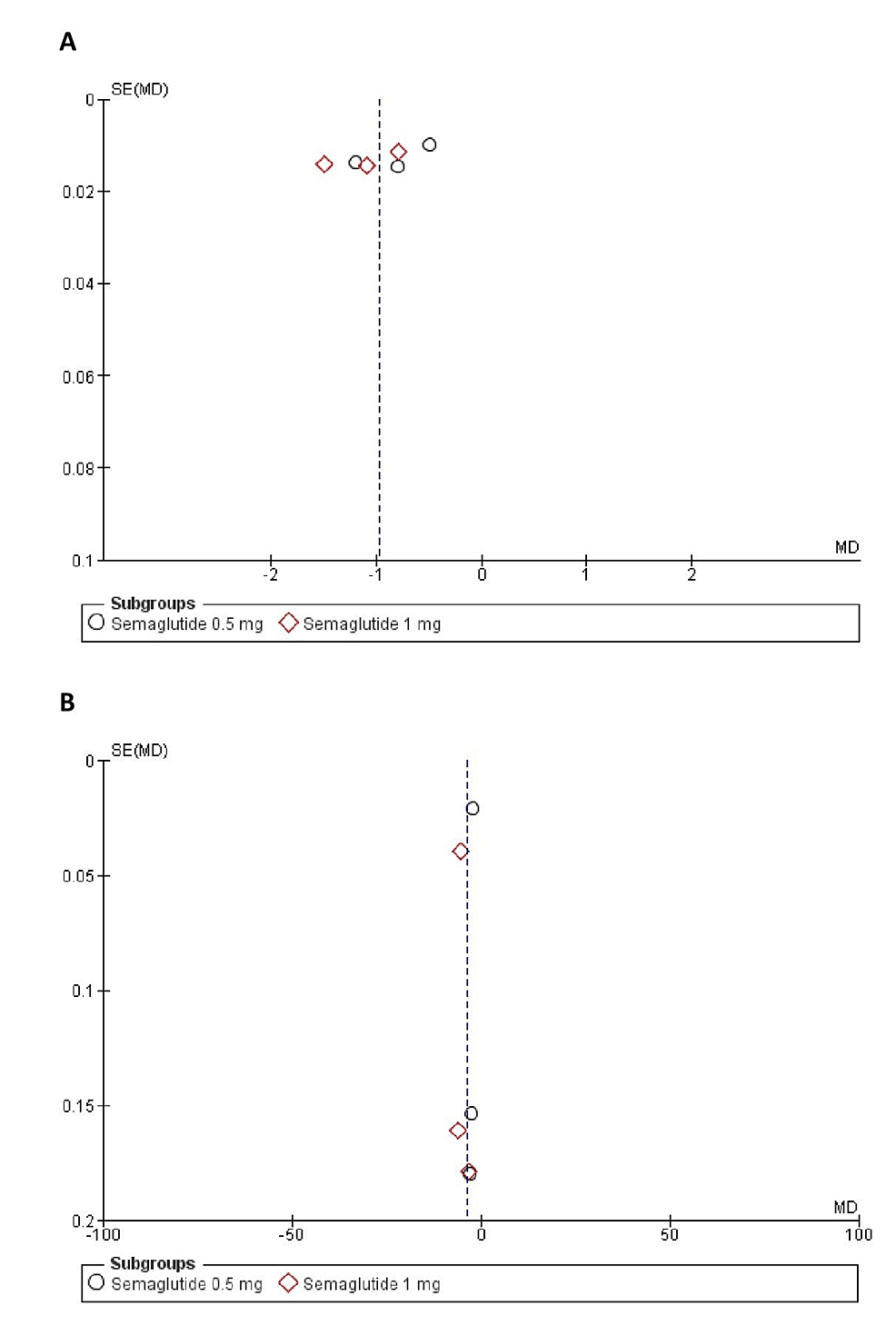


HBA1C: Glycated hemoglobin, SBP: Systolic blood pressure, SE: Standard error, WMD: Weighted mean difference, RR: Relative risk

**Figure S3: Funnel plots: (A) Changes in DBP (B) Change in Pulse rate**


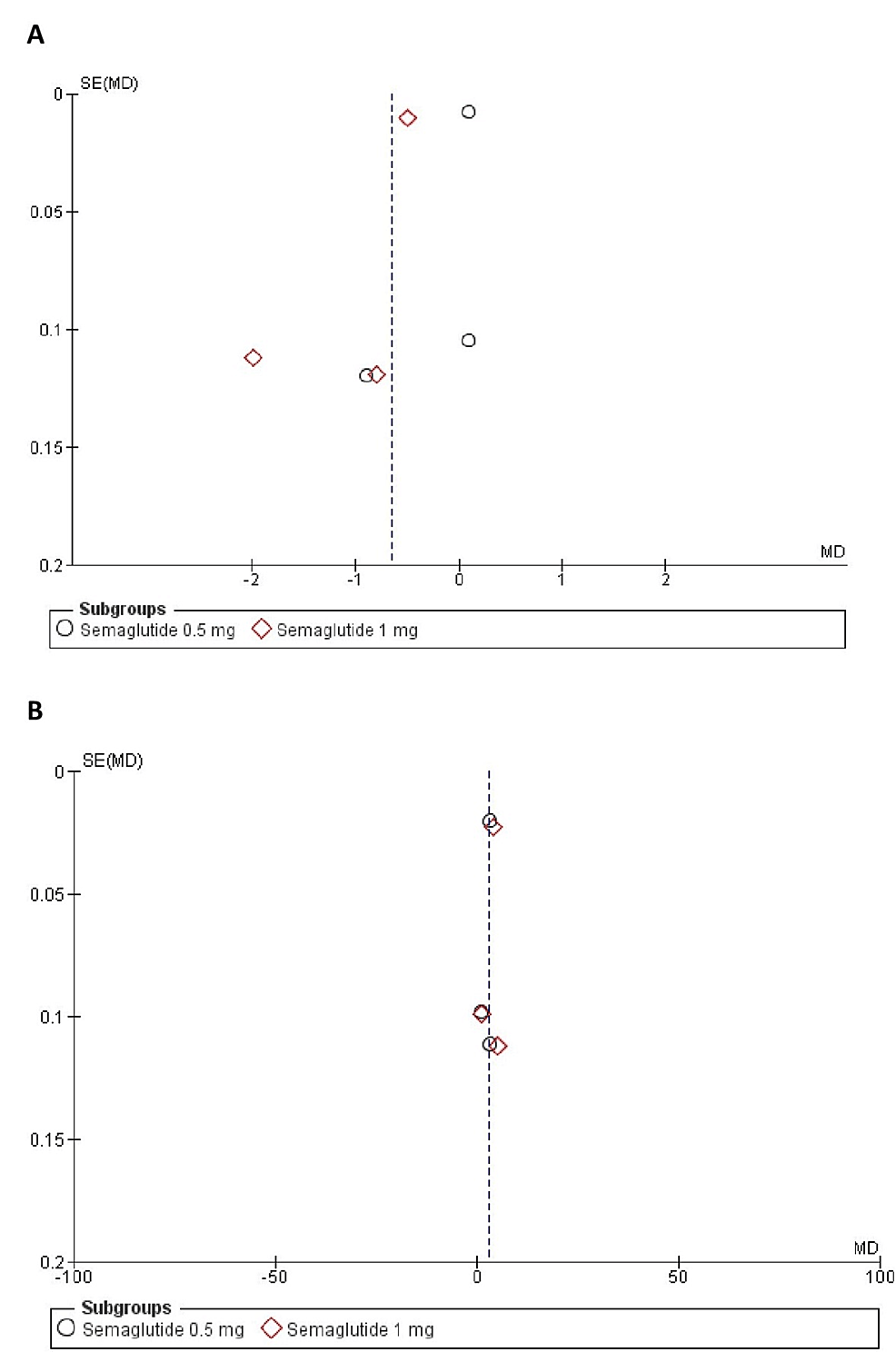


DBP: Diastolic blood pressure, SE: Standard error, WMD: Weighted mean difference, RR: Relative risk

**Figure S4: Funnel plots: (A) Change in Body weight (B) Change in Waist circumference**


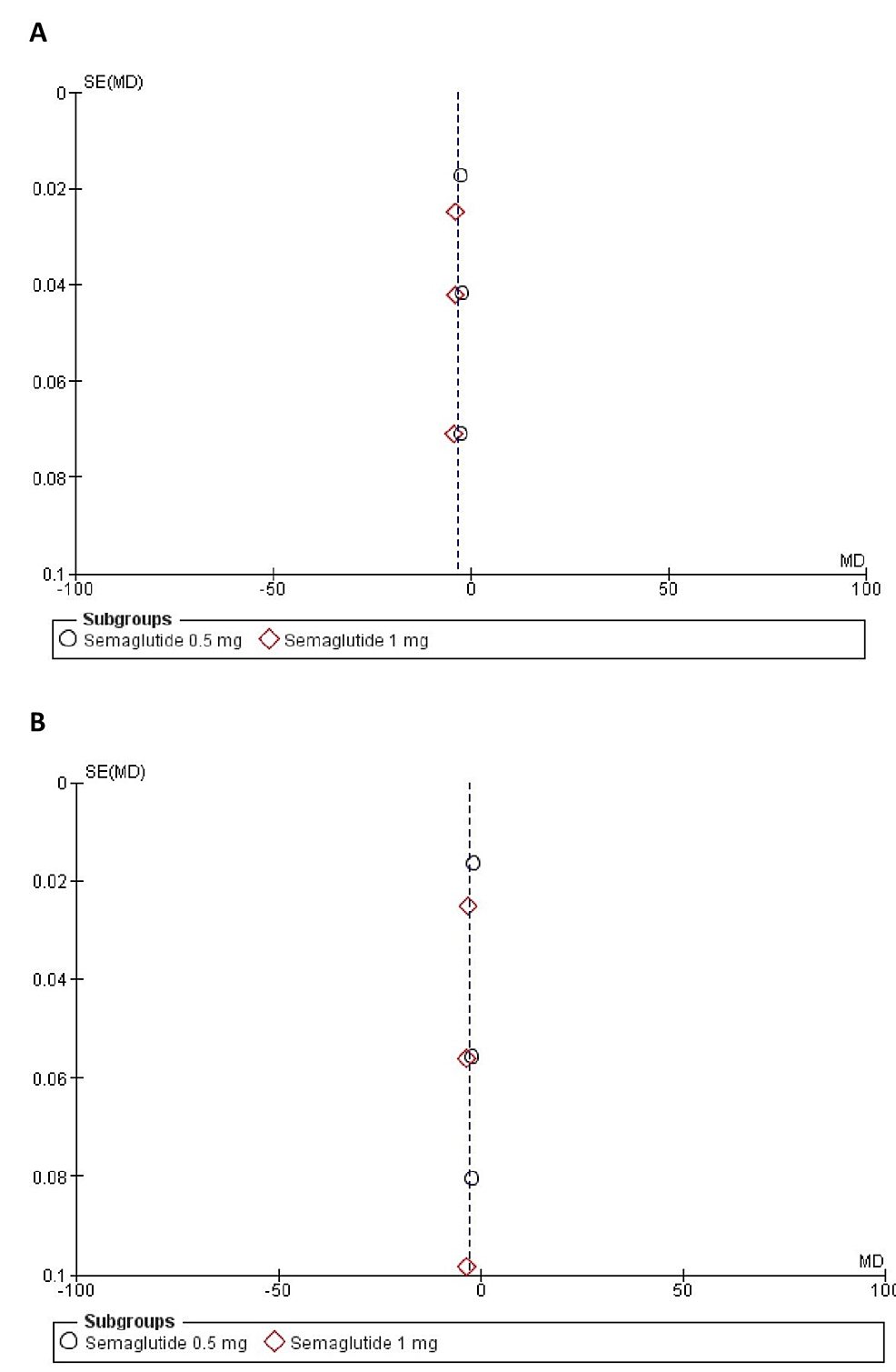


SE: Standard error, WMD: Weighted mean difference, RR: Relative risk

**Figure S5: Funnel plots representing BMI**


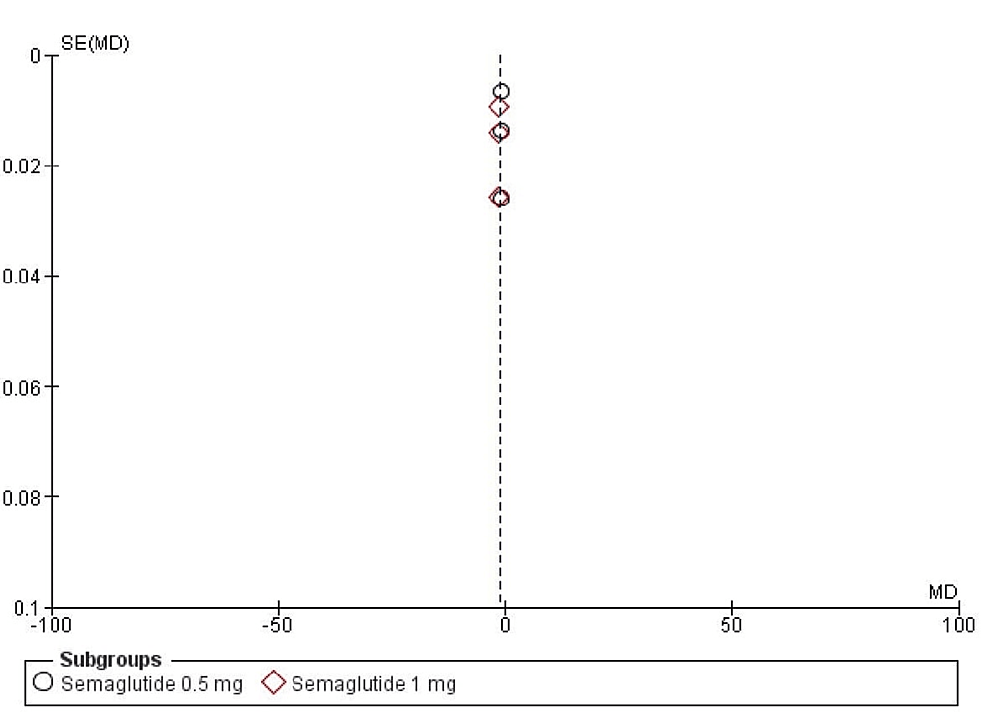


BMI: Body mass index, SE: Standard error, WMD: Weighted mean difference, RR: Relative risk
