# Supplementary material for: Comparative efficacy and safety profile of once-weekly Semaglutide versus once-daily Sitagliptin as an add-on to metformin in patients with type 2 diabetes: a systematic review and meta-analysis
Source: Ann Med. 2023 Jul 27;55(2):2239830. doi: 10.1080/07853890.2023.2239830 (PMC10375936; doi:10.1080/07853890.2023.2239830)
Supplement: Supplemental Material [file IANN_A_2239830_SM4748.docx]

| Database | Search strategy | Results |
| --- | --- | --- |
| PubMed | ("semaglutide"[Supplementary Concept] OR "semaglutide"[All Fields]) AND ("sitagliptin phosphate"[MeSH Terms] OR ("sitagliptin"[All Fields] AND "phosphate"[All Fields]) OR "sitagliptin phosphate"[All Fields] OR "sitagliptin"[All Fields] OR "sitagliptine"[All Fields]) AND ("once"[All Fields] AND "weekly"[All Fields]) AND ("once"[All Fields] AND ("dailies"[All Fields] OR "daily"[All Fields])) AND ("randomized controlled trial"[Publication Type] OR "randomized controlled trials as topic"[MeSH Terms] OR "randomized controlled trial"[All Fields] OR "randomised controlled trial"[All Fields]) AND (("safety"[MeSH Terms] OR "safety"[All Fields] OR "safeties"[All Fields]) AND ("efficacies"[All Fields] OR "efficacious"[All Fields] OR "efficaciously"[All Fields] OR "efficaciousness"[All Fields] OR "efficacy"[All Fields])) | 5 |
| Cochrane library | semaglutide AND sitagliptin AND once weekly AND once daily AND randomized controlled trial AND safety and efficacy | 10 |
| Elsevier’s ScienceDirect | semaglutide AND sitagliptin AND once weekly AND once daily AND randomized controlled trial AND safety and efficacy | 10 |
